# PAR-Complex and Crumbs Function During Photoreceptor Morphogenesis and Retinal Degeneration

**DOI:** 10.3389/fncel.2018.00090

**Published:** 2018-03-29

**Authors:** Franck Pichaud

**Affiliations:** Medical Research Council, Laboratory for Molecular Cell Biology, University College London, London, United Kingdom

**Keywords:** cell polarity, epithelial cells, sensory neurons, retina, Crumbs complex, PAR complex, *Drosophila melanogaster*, neuroepithelial cells

## Abstract

The fly photoreceptor has long been used as a model to study sensory neuron morphogenesis and retinal degeneration. In particular, elucidating how these cells are built continues to help further our understanding of the mechanisms of polarized cell morphogenesis, intracellular trafficking and the causes of human retinal pathologies. The conserved PAR complex, which in flies consists of Cdc42-PAR6-aPKC-Bazooka, and the transmembrane protein Crumbs (Crb) are key players during photoreceptor morphogenesis. While the PAR complex regulates polarity in many cell types, Crb function in polarity is relatively specific to epithelial cells. Together Cdc42-PAR6-aPKC-Bazooka and Crb orchestrate the differentiation of the photoreceptor apical membrane (AM) and *zonula adherens (ZA)*, thus allowing these cells to assemble into a neuro-epithelial lattice. In addition to its function in epithelial polarity, Crb has also been shown to protect fly photoreceptors from light-induced degeneration, a process linked to Rhodopsin expression and trafficking. Remarkably, mutations in the human *Crumbs1* (CRB1) gene lead to retinal degeneration, making the fly photoreceptor a powerful disease model system.

## Introduction

The fly retina is a very popular model system that has been used to study a wide range of biological processes. This popularity is due to its intermediate level of complexity with regards to cell specification and morphogenesis and its excellent genetic tractability. Following many decades of work on this organ, our knowledge of how photoreceptor neurons and their accessory cells are specified is very comprehensive. Photoreceptors are specialized sensory neurons that are born in the eye imaginal disc, which consists of epithelial cells that have been primed to differentiate into retinal cells through an eye specific regulatory gene network that includes the master eye regulator *eyeless/pax6* (Casares, 2016). During their early phase of differentiation, photoreceptors assemble into basic units called ommatidia and initiate neurogenesis (Ready et al., 1976; Cagan and Ready, 1989; Wolff and Ready, 1991). Neurogenesis in these cells includes the specification of the axon that navigates through the optic stalk toward the developing optic lobe.

**Figure 1:**
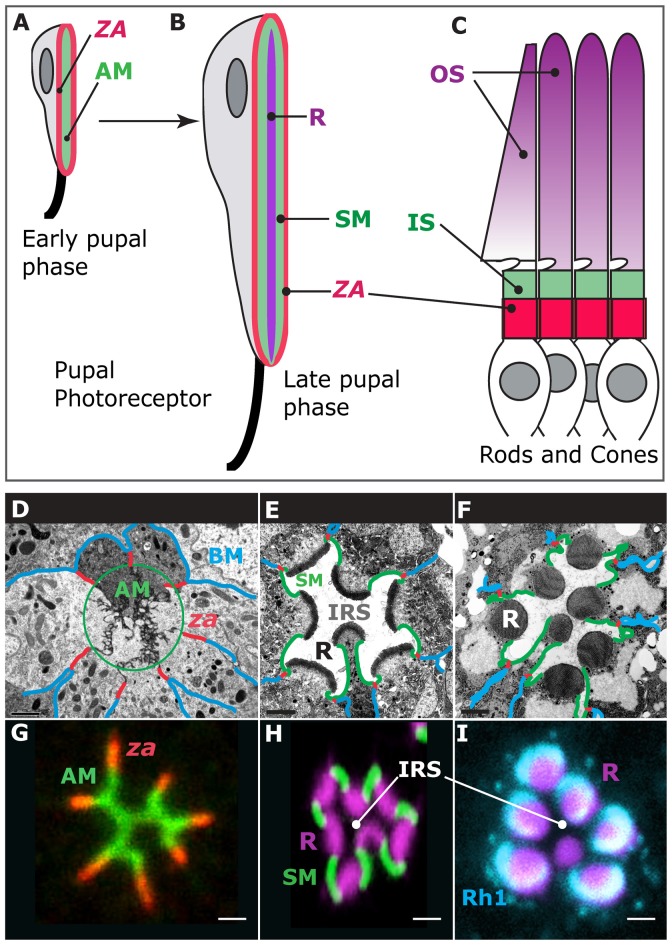
Photoreceptor morphogenesis. Early stage of pupal photoreceptor morphogenesis showing the *zonula adherens* (*ZA*, red) and the nascent apical membrane (AM; green). **(B)** Late pupal photoreceptor presenting a mature Rhabdomere (R, purple), Stalk membrane (green) and *ZA* (red). From **(A,B)**, the pupal photoreceptor has extended along the proximal (top)-distal (bottom) axis. **(C)** Vertebrate photoreceptors (cone and rods). The outer segment (OS, purple) is supported by the inner segment (IS, green). The *ZA* is shown in red. **(D–F)** Electron micrographs of representative stages of pupal photoreceptor development. Stages of pupal photoreceptor development can be expressed as a function of the % of pupal development, where 0% marks the onset of pupal life and 100% mark the adult animal from the pupal case. **(D)** Early pupal ommatidium (40%) where seven photoreceptors can be seen. Basal membrane (BM) is in blue, the *ZA* in red and the AMs are circled (green). **(E)** 65% pupal ommatidium where the stalk membrane (SM, green) and Inter rhabdomeric space (IRS) are clearly visible. R stands for rhabdomere. **(F)** Adult ommatidium showing mature rhabdomeres (R), stalk membranes (green), *ZA* (red) and lateral membranes (blue). **(G)** 40% ommatidium stained for aPKC (green), which labels the AMs and the stained for *ZA* marker Armadillo (red). **(H)** 60% ommatidium stained for F-actin using phalloidin (purple), which predominantly labels the developing rhabdomere (R), and Crumbs (green), labeling the stalk membranes. IRS stands for Inter-Rhabdomeric-Space. **(I)** Mature ommatidium (90% after puparium formation) stained for F-actin (purple) and Rh1 (turquoise).

Photoreceptor morphogenesis unfolds during the pupal life of the animal as these cells repolarize their apical (top)-basal (bottom) axis and evolve new membrane domains, including the rhabdomere, which is the light-gathering organelle of the cell (Ready, 2002; Figures 1A,B,F). The rhabdomere is an enormously amplified apical membrane (AM) that contains approximately 60,000 microvilli (Arikawa et al., 1990; Figure 1F) that bear the visual pigment, Rhodopsin (Figure 1I), and the signalplex, which is a hub for phototransduction (Wang and Montell, 2007). The rhabdomere is supported by a specialized membrane, called the stalk membrane, which lies immediately apical to the cell’s *zonula adherens* (*ZA*; Ready, 2002; Figures 1B,E,F,H), and can be compared to the inner segment (IS) that supports the outer segment (OS) in vertebrate cone and rod photoreceptors (Figures 1B,C). In epithelial cells, the* ZA* contains the adhesion molecule E-Cadherin and is the main intercellular adhesion domain that allows cells to assemble into sheets to form organs (Tepass, 2012). In the pupal photoreceptor, differentiation of the AM and *ZA* takes place early during pupal development, and both membrane domains are readily visible by 37% after puparium formation (Figures 1A,D,G; where 0% is the animal entering pupation and 100% corresponds to the eclosion of the fly). Later on, at around 55% after puparium formation, the stalk membrane is clearly visible and distinct from the apical microvilli and *ZA* (Figures 1B,D,G; Longley and Ready, 1995). Toward the end of pupal life, at approximately 75% after puparium formation, transcription of the *rhodopsin* genes is activated and Rhodopsins populate the rhabdomere, contributing to its final phase of morphogenesis and maintenance (Figures 1F,I; Kumar and Ready, 1995; Chang and Ready, 2000; Pinal and Pichaud, 2011). The genetic tractability of the pupal photoreceptor, together with its large size and exquisite accessibility for imaging makes it an ideal model system to study sensory neuron morphogenesis. In particular, the pupal photoreceptor has proven very valuable to study the mechanisms of epithelial polarity and the regulatory networks involved in specifying and building the epithelial *ZA*. In addition, the fly photoreceptor has also been very powerful in studying disease genes that in vertebrates cause retinal degeneration.

## Regulatory Networks Driving Polarized Morphogenesis

Work in the *C. elegans* zygote has led to the discovery of the PARtitioning defective genes (PAR1–6), which when mutated lead to defects in setting up the antero-posterior axis of the zygote (Goldstein and Macara, 2007). Remarkably, this regulatory network is largely conserved through evolution and is used to regulate polarity in epithelial and neuroepithelial cells, as well as in neurons.

### Cdc42 Orchestrates Photoreceptor Morphogenesis Through the PAR Complex and Pak4

**Figure 2:**
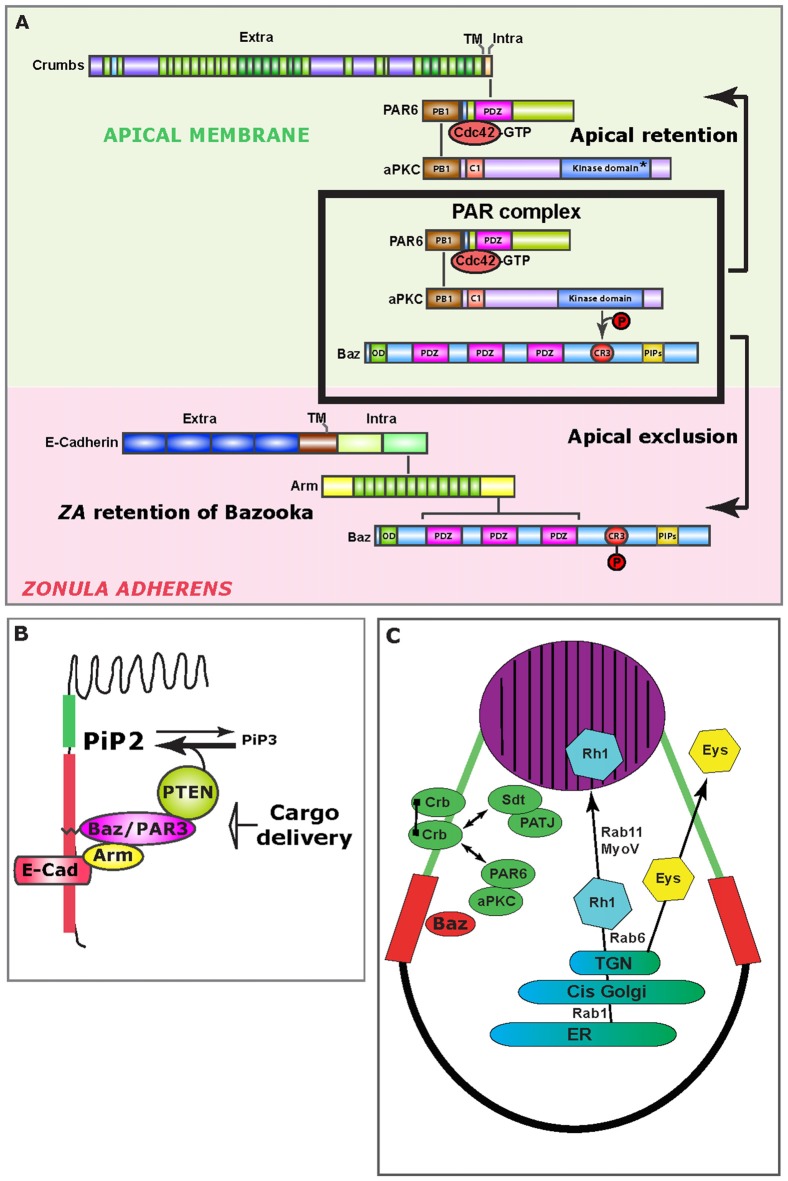
Mechanisms of epithelial polarity. **(A)** Upon PAR complex assembly (boxed), aPKC phosphorylates Bazooka (Baz), which leads to the apical exclusion of PS980-Baz and concomitant apical retention of Cdc42-PAR6-aPKC, which is enabled through Crumbs (Crb) at the apical/stalk membrane (green background). At the developing *ZA* (pink background), Bazooka is retained, presumably through direct binding to Armadillo. **(B)** Schematic representation of how the Baz-PTEN association contributes in limiting apical/*ZA* levels of PiP3. Preventing the accumulation of PiP3 at the photoreceptor AM is important for the specification of the rhabdomere. **(C)** Schematic representation of the trafficking route that supports delivery of Rhodopsin 1 (Rh1) to the rhabdomere (purple). Potential cis-interactions between Crb molecules are represented by a line joining two Crb molecules at the stalk membrane (green). Double-sided arrows represent protein interactions. The *ZA* is shown in red. ER, Endoplasmic Reticulum; TGN, Trans Golgi network.

At the core of the epithelial polarity protein regulatory network that governs pupal photoreceptor morphogenesis is the small GTPase Cdc42. Cdc42 belongs to the large family of Rho-GTPases and can be activated (Cdc42-GTP) at specific sub-cellular locations through Guanine nucleotide Exchange Factor (GEFs) or inactivated (Cdc42-GDP) by GTPase-Activating Proteins (GAPs; Hall, 2012). Spatial activation/inactivation of Cdc42 has been shown to act as a conserved polarity mechanism from budding yeast to human cells (Etienne-Manneville, 2004; Park and Bi, 2007). Work in the *C. elegans* embryo supports the notion that spatial distribution of GEFs or GAPs might dictate where the PAR complex assembles (Anderson et al., 2008; Chan and Nance, 2013; Klompstra et al., 2015). In these cells, Cdc42 is inactivated at the lateral cell contacts through the Cdc42 GAP PAC-1 and is activated at the contact free membrane by two GEF, ECT-2 and CGEF-1, thus promoting PAR complex assembly. However, spatial regulation of Cdc42 does not necessarily operate in all epithelial tissues in *C. elegans* (Zilberman et al., 2017). In pupal photoreceptors, and in epithelial cells in general, active Cdc42 is essential for the assembly of the PAR complex at the nascent AM (Joberty et al., 2000; Lin et al., 2000; Hutterer et al., 2004; Walther and Pichaud, 2010; Jin et al., 2015; Figure 2A). Therefore, it is tempting to speculate that spatially restricted activation of Cdc42 could act as a polarization mechanism in epithelial cells by allowing localized PAR complex assembly and signaling.

In pupal photoreceptors, Cdc42 function during polarized morphogenesis is mediated by two main effectors: (i) the PAR complex; and (ii) the conserved p21-activated kinase 4 (Pak4/Mbt).

#### Cdc42 and the PAR Complex

As the Cdc42-PAR6-aPKC-Bazooka complex assembles, Bazooka (Baz) is phosphorylated by aPKC on the conserved Serine 980 (S827 in vertebrate PAR3), and this phosphorylation allows for the separation of PS980-Baz from Cdc42-PAR6-aPKC, enabling signaling through aPKC (Hirose et al., 2002; Nagai-Tamai et al., 2002; Krahn et al., 2010; Morais-de-Sá et al., 2010; Walther and Pichaud, 2010; Figure 2A). All indications are that while Baz is required for the loading of the PAR complex at the plasma membrane, its phosphorylation by aPKC is constitutive. Breaking up the PAR complex into two products (Cdc42-PAR6-aPKC and PS980-Baz) acts as the main symmetry-breaking mechanism that drives the separation of the *ZA* from the AM. This mechanism is based on the selective retention of Cdc42-PAR6-aPKC and concomitant exclusion of PS980-Baz from the developing AM. However how this is achieved is not fully understood. Following apical exclusion, PS980-Baz accumulates at the border between the apical and lateral membrane (Figure 2A) where it is thought to regulate *ZA* assembly via recruiting *adherens junction* material (Wei et al., 2005; McGill et al., 2009; Figure 2A). PAR complex signaling mediated by aPKC is conserved through evolution and plays a role during vertebrate epithelial and neuroepithelial cell polarization, as well as axon specification in cortical neurons (Goldstein and Macara, 2007; St Johnston and Ahringer, 2010; Kon et al., 2017).

#### Cdc42 and p21-Activated Kinase 4

Another function for Cdc42 in developing the pupal photoreceptor is to promote retention of PS980-Baz at the developing *ZA*. For this retention mechanism, Cdc42 functions through the oncogene p21-activated kinase Pak4/Mbt (Walther et al., 2016). In human epithelial cells, Pak4 functions downstream of Cdc42 to promote tight junction and *adherens junction* maturation (Wallace et al., 2010; Jin et al., 2015). In flies, Mbt phosphorylates the *adherens junction* protein Armadillo/β-Catenin at two conserved Serine residues (Menzel et al., 2008), a function also reported in Zebrafish (Selamat et al., 2015). In pupal photoreceptors, Armadillo phosphorylation by Mbt/Pak4 regulates the retention of Baz at the developing *ZA* and promotes its morphogenesis (Schneeberger and Raabe, 2003; Menzel et al., 2007, 2008). In these cells, retention of Baz functions redundantly with PAR1-dependent lateral displacement (Benton and St Johnston, 2003; McKinley and Harris, 2012; Walther et al., 2016), so to prevent Baz from accumulating at the lateral membrane, where it could recruit ectopic aPKC and *adherens junction* material. *ZA* retention of Baz is therefore an essential mechanism that contributes to enhancing polarization of the cell along the apical-basal axis (Figure 2A). Whether a similar junctional retention mechanism operates in vertebrate cells to prevent lateral spreading of the PAR complex remains to be investigated.

### Polarized Accumulation of Phosphoinositol Lipids Regulates Apical Membrane Morphogenesis

Amongst the many examples for the utility of the fly pupal photoreceptor in studying polarized cell morphogenesis is the discovery that spatially restricted accumulation of phosphoinositol lipids regulates AM morphogenesis (Pinal et al., 2006; Figure 2B). Pioneering work in the fly embryonic ectoderm revealed that Bazooka can bind and recruit the lipid phosphatase PTEN at the apical pole of epidermis cells (von Stein et al., 2005). PTEN catalyzes the conversion of PiP3 into PiP2 (Leslie and Downes, 2002), and its apico-lateral recruitment correlates with local accumulation of PiP2 (von Stein et al., 2005). Concomitant with this study, we reported that in the pupal photoreceptor, PTEN recruitment by Bazooka at the developing *ZA* puts a break on the apical levels of PiP3 and promotes PiP2 enrichment at the AM and* ZA* (Pinal et al., 2006). This regulation is important because when it is abolished in PTEN mutants, stalk membrane is ectopically inserted within the apical rhabdomere. Subsequent studies in vertebrate MDCK cells showed the interaction between PAR3 and PTEN to be conserved and suggested that such defects during polarized plasma membrane morphogenesis could be explain by a role for polarized PiP2/PiP3 accumulation in directing trafficking (Bryant and Mostov, 2008). In addition, a link between Baz/Par3 and PTEN suggests that in epithelial cells, loss of polarity or adhesion might directly impact on cell growth through the Serine/Threonine kinase Akt, which is activated by PiP3 (Kim et al., 2017).

### Crumbs and Positive Feedback Loops During Epithelial Cell Polarization

For polarity to arise, molecular asymmetries must be established at the plasma membrane that are reinforced through feedback loops (Altschuler et al., 2008; Goryachev and Pokhilko, 2008; Lo et al., 2014). In the pupal photoreceptor, the transmembrane protein Crumbs (Crb) is an important player during polarized morphogenesis because it recruits Cdc42-PAR6-aPKC at the nascent stalk membrane, thus re-enforcing the molecular asymmetry created by the PAR complex. Crb contains a large extra-cellular domain that can mediate homophilic adhesion (Tepass et al., 1990; Wodarz et al., 1995; Roper, 2012; Zou et al., 2012; Letizia et al., 2013). This protein also contains a short intracellular domain that is sufficient to promote polarity in many epithelia by recruiting a set of proteins, including the polarity regulators Stardust, and also PAR6/aPKC (Wodarz et al., 1995; Klebes and Knust, 2000; Bachmann et al., 2001; Hong et al., 2001; Lemmers et al., 2004). Early work using the pupal photoreceptor showed that PAR6 can bind to the short PDZ-binding domain of Crb via its conserved PDZ domain (Nam and Choi, 2003). Subsequent work showed that Crb recruitment of Cdc42-PAR6-aPKC is required for the separation of the *ZA* from the stalk membrane (AM in other cell types; Walther and Pichaud, 2010; Figure 2A). Accordingly, *crb* mutant photoreceptors fail to build their *ZA* (Izaddoost et al., 2002; Pellikka et al., 2002). In binding to PAR6/aPKC, Crb likely stabilizes these proteins at the AM and contributes to sustaining the production of Cdc42-PAR6-aPKC and PS980-Baz, which in turn drives polarized morphogenesis of the plasma membrane. It is interesting to note that the topology of the pupal photoreceptor and other epithelial fly cells most closely resembles that of vertebrate neuroepithelial cells (Aaku-Saraste et al., 1996; Chenn et al., 1998), in that the apical junction consists mostly of Cadherin, and the regulatory network discussed in this review plays an important role in regulating polarity in these cells (Afonso and Henrique, 2006). It is therefore conceivable that during brain development in vertebrates, deregulation of this network might lead to defects in neuroepithelial cell polarity and adhesion that could cause pathologies.

## The Fly Photoreceptor as a Disease Model

Over the past few decades the fly photoreceptor has been used as a powerful model system to study human retinopathies, including those linked to defects in Rhodopsin trafficking. Examples of its relevance to study human diseases can be found in very elegant studies on Crb function in preventing light-induced retinal degeneration.

### Crumbs and Retinal Degeneration

Next to its function during polarized morphogenesis, Crb is required to protect photoreceptors from light induced degeneration (Johnson et al., 2002). This is interesting because mutations in one of the* crb* human orthologs, *CBR1*, lead to retinitis pigmentosa (RP12) and Leber Congenital Amaurosis (LCA), which are inherited retinopathies characterized by photoreceptor degeneration and blindness (Richard et al., 2006; den Hollander et al., 2008).

Three *crb* genes are found in humans that encode isoforms of the *CRB1–3* proteins. *CRB1* and *CRB2* are most similar to *Drosophila* *crb*, while *CRB3* encodes for a shorter version that lacks the large extracellular domain. CRB1 is expressed in the mammalian retina and localizes at the inner photoreceptor segment (Figure 1C) as well as apical to the *adherens junction* of Muller cells, which are retinal glial cells that support photoreceptor function. Up to 150 mutations have been identified in *CRB1* that cause retinopathies (Bujakowska et al., 2012), a majority of which are located in the large extracellular domain of the protein. The large extracellular domain of Crb is not strictly required for polarity in some epithelia (e.g., fly embryonic epidermis), but it is required alongside the intracellular domain for proper pupal photoreceptor morphogenesis (Richard et al., 2006; Pellikka and Tepass, 2017). In addition, the extracellular domain of Crb carries functions that can be dissociated from Crb role in polarized morphogenesis. This includes a function in regulating Notch signaling in flies and zebrafish (Herranz et al., 2006; Richardson and Pichaud, 2010; Ohata et al., 2011; Nemetschke and Knust, 2016), as well as a role in promoting cell sorting. This is the case in the fish retina, where two Crb isoforms, CRB2a and CRB2b are expressed and play a role in both photoreceptor *ZA* morphogenesis and clustering of cone photoreceptors, possibly though CRB-CRB trans-interaction mediated adhesion (Zou et al., 2012).

Crb-Crb interaction mediated by the extracellular domain is conserved from flies to vertebrates (Roper, 2012; Letizia et al., 2013) and might play a role in protecting photoreceptors from light-induced stress. In this context, the fly photoreceptor has allowed for very significant advances in our understanding of how Crb/CRB1 might promote photoreceptor survival when challenged using light. Firstly, light induced degeneration in *crb* mutant photoreceptors can be significantly suppressed when the flies are fed a diet containing low levels of vitamin A (carotenoid), which limits the production of Rhodopsin (Johnson et al., 2002). Secondly, Crb has been shown to regulate Rhodopsin trafficking. In flies, Crb interacts with the motor protein MyosinV, which together with the small GTPase Rab11 and its regulator dRip11 regulates trans-Golgi to plasma membrane trafficking of Rhodopsins (Satoh et al., 2005; Li et al., 2007; Figure 2C). In this trafficking pathway, Rab11 regulates ER to Golgi transport (Satoh et al., 2005), while recent work revealed that Rab6 specifically regulates sorting of apical cargo, including Rhodopsin, from the trans-Golgi (Iwanami et al., 2016). Thirdly, recent studies in which *crb* was mutagenized at conserved residues located in the extracellular domain that are associated with human retinopathies, have revealed a complex pattern of trafficking for Crb, and how it might in turn influence Rhodopsin trafficking, including endocytosis (Lin et al., 2015; Pellikka and Tepass, 2017). Finally, recent work indicates that alternative splicing variants of the crb locus are expressed in flies, and that in the retina a particular isoform (Crb-C) is especially relevant for preventing light-induced degeneration (Spannl et al., 2017). Defects in Rhodopsin trafficking in humans are a major cause of retinal dystrophy, and work in flies strongly suggests that it is, at least in part, defects in Rhodopsin trafficking that cause light induced degeneration in *crb* mutant photoreceptors.

In addition, Crb functions as part of a regulatory network that promotes rhabdomere morphogenesis and inter rhabdomeric space (IRS) formation (Gurudev et al., 2014). Included in this network are the secreted proteins Prominin1 (Prom1) and the conserved proteoglycan Eyes shut (Eys; Figure 2C), both of which have orthologs that are linked to human retinal dystrophies when mutated (Maw et al., 2000; Husain et al., 2006; Zelhof et al., 2006; Collin et al., 2008; Gurudev et al., 2013).

## Concluding Remarks

The fly photoreceptor has continued to be a superb model system in which to discover and study the mechanisms that regulate neuronal differentiation, morphogenesis and physiology. Despite the tremendous advances in gene editing in vertebrate models and our ability to produce organoids *in vitro*, the *Drosophila* retina remains a very attractive model system to study the cell and molecular biology of tissue development and function* in vivo*, because it comes equipped with a very versatile genetic tool-box combined with live imaging and low gene redundancy. It also comes with a wealth of knowledge that is not found in many other systems, in turn allowing for the generation of a much more integrated view of specific cell and biological processes *in vivo*. The full mechanisms of epithelial and neuronal polarity establishment remain elusive and more work is required to truly understand how polarity arises in these main cell types. Whether polarity can be generated through spatial regulation of Cdc42 is an exciting possibility, but what mechanisms are responsible for localizing the relevant GEFs/GAPs? What are the biophysical properties of the PAR complex that might explain the mechanisms that promote its partitioning, which in fly epithelial cells promotes separation of the *ZA* from the AM? How does membrane delivery come together with the PAR complex to promote polarized plasma membrane morphogenesis? Alongside other well studied epithelial cell types, the pupal photoreceptor will continue to yield insights into all of these questions, which are fundamental to our understanding of epithelial and neuron morphogenesis.

## Author Contributions

FP wrote the manuscript and prepared the Figures.

## Conflict of Interest Statement

The author declares that the research was conducted in the absence of any commercial or financial relationships that could be construed as a potential conflict of interest.
